# Investigation of the Awareness of the Students of Shiraz Dental School Concerning the Patients’ Rights and the Principles of Ethics in Dentistry

**Published:** 2013-03

**Authors:** SZ Tabei, MR Azar, F Mahmoodian, N Mohammadi, H Farhadpour, Y Ghahramani, Z Al-e-Yasin

**Affiliations:** a Dept. of Medical Ethics, School of Medicine, Shiraz University of Medical Sciences, Shiraz, Iran; b Dept. of Endodontic, School of Dentistry, Shiraz University of Medical Sciences, Shiraz, Iran; c Dept. of Medical Ethics, Faculty member and research vice-chancellor, Shiraz University of Medical Sciences, Shiraz, Iran; d Dept. of Pediatric Dentistry, School of Dentistry, Shiraz University of Medical Sciences, Shiraz, Iran; e Dept. of Restorative Dentistry, School of Dentistry, Shiraz University of Medical Sciences, Shiraz, Iran; f Dept. of Endodontics, School of Dentistry, Shiraz University of Medical Sciences, Shiraz, Iran; g Dept. of Oral Medicine, School of Dentistry, Shiraz University of Medical Sciences, Shiraz, Iran

**Keywords:** Patients’ Rights, Dentistry, Professional Ethics

## Abstract

**Statement of Problem: **Deliberating the patients’ rights is one of the major human ethical and legal principles which can be investigated through the agenda of professional and medical ethics.accordingly , the students of dentistry have to be educated about this issue and achieve the necessary skills in deliberating and concerning the patients’ rights.

**Purpose: **The present study aimed to investigate Shiraz dental students’ awareness and attitude regarding the patients’ rights as well as the principles of dental ethics in order to design methods for organizing and improving the ethics in dentistry.

**Materials and Method:** The present descriptive-analytical study was conducted on 111 students of different departments of Shiraz Dental School. The study data were collected through a questionnaire designed based on the patients’ rights charter in Iran, ADA ethical codes which are internationally acceptable in the field of dentistry, and the guidelines of the ministry of health, treatment, and medical education (No. 140588). Then, the data were entered into the SPSS statistical software and analyzed.

**Results:** Of all participants, 21.6% were men and 78.4% were women. According to the results, 71% of the students were acquainted with the patients’ rights. In addition, the patients’ characteristics, including gender and nationality, were not important for 58.6% of the students.

**Conclusion:** The study findings showed that 71% of the participants were acquainted with the patients’ rights Establishment of a comprehensive, integrated charter in Iran is needed. With rapid development of medical sciences, new issues appear which necessitate taking into account the mutual rights of the physicians, people, and patients.

## Introduction

Medical ethics is defined as a structured system which aims to present appropriate strategies for solving the ethical problems in medical sciences and dentistry [1]. In all countries, laws are endorsed for adjusting the performance of health specialists. In fact, these laws have been enacted in order to support the members of the society against the inefficient specialists and, at the same time, accurately describe the performance of the qualified ones [2]. Emphasis on the human rights in healthcare, particularly respecting the patients’ dignity, is of utmost importance in particularly when patients’ vulnerability exposes them to the violations [3].

Nowadays, improvements in knowledge and technology in prevention, diagnosis, and treatment have provided the physicians with a large number of decisions which has, consequently, resulted in creation of new issues which cannot be encountered by Hippocratic medical ethics. Therefore, teaching the imperious and unfriendly ethical principles is not sufficient and the students must be trained regarding new ethical strategies for solving the problems [4].

Today, the patients’ rights consist of both individual and social aspects. More emphasis has been put on care and treatment rights, sufficient social coverage, proper information on healthcare services, and the right to utilize the social developments. Moreover, economic signs, modern medicine, and patients’ expectations all affect the identification of the patients’ rights. Thus, it is quite necessary to develop principles to support the individuals in healthcare with emphasis on the patients’ rights [5].

Fesharaki, Tofighi, and Nematollahi conducted a study and revealed that no integrated set of patients’ rights existed in Iran [6].

Salimi and Yarmohammadian also performed a study and showed that the rate of knowledge and observation of the patients’ rights charter, concerning the patients’ dignity and observing the supporting performances was above the average level [7].

The present study aims to investigate Shiraz dentistry students’ awareness and attitude toward this issue and, at the same time, assess their ethical performance in order to design methods for organizing and improving the observation of dental ethics, particularly regarding the patients’ rights.

## Materials and Method

The present descriptive-analytical, cross-sectional study was conducted on a number of Shiraz Dentistry School students who had passed their basic courses. The students’ opinions were collected by providing questionnaire which was designed on the basis of patients’ rights charter in Iran, American Dental Association (ADA) ethical codes, and the guidelines of the ministry of health regarding the principles of infection control. Then, the questionnaire was given to the experts and professors in the related field and its validity was confirmed. It was also given to twenty students in a pilot study and its reliability was confirmed (Cronbach’s alpha=0.7). Finally, the questionnaire was given to the students who were willing to take part in the research. All the questions of the questionnaire were closed ones. With exception for some individual characteristics, all the variables were qualitative type and were reflecting the opinions of the participants regarding the patients’ rights and ethical principles and the rate of complying with them. Collected data were entered into the SPSS statistical software and analyzed. The following is the selection of the principles employed in designing the questions derived from ADA:

- Section1: Principle: Patient autonomy (self-governa-nce)

1. A: Patient involvement 

1. B: Patient records 

1. B.2: Confidentiality of patient records

- Section 2: Principle: Non-maleficence (do no harm)

2. A: Education 

2. B: Consultation and referral 

2. C: Use of auxiliary personnel 

2. D.1: Ability to practice 

2. E: Exposure to blood-borne pathogen 

2. G: Personal relationship with patient

- Section 3: Principle: Beneficence (do Well)

3. A: Community service

- Section 4: Principle: justice (fairness)

4. A: Patient selection 

4. A.1: Patient with blood-borne pathogen

4. B: Emergency service

4. C: Justifiable criticism

4. D: Expert testimony

5. A.2: Unsubstantiated representations 

5. B: Representation of fees [8] 

## Results

Among the 111 participants of the present study, 21.6% were male and 78.4% were female individuals. According to the results, 35.1% of the students stated that they can sometimes misrepresent the dangers of refusing the treatment for the patients (5.A.2). Based on the principle of “community service” (3.A.), dentists have to use their skills, knowledge, and experience to improve the society’s dental health. The correct responses to this question were 58.8%. 

In case of suffering from diseases, such as hepatitis, which would affect the treatment process, 76.6% of the students mentioned that they would limit their work such a way that it would have no effects on the treatment (2.D.1). In this study, 53.2% of the students showed motivation to train and cooperate with an individual as auxiliary personnel (2.C).

54.1% of the students mentioned that they orally got the patients’ permission to pass their information to another dentist and 58.6% of the participants stated that the patients’ characteristics, such as gender and nationality, were not important for them (4.A).

Moreover,85.6% of the study participants believed in informing the patients about their oral and dental status without giving comments on the previous dentist’s performance (4.C). Likewise, 61.3% of the students declared that they completely explained the treatment processes to the patients (1.A) and 83.8% of the participants claimed that they informed the patients about the treatment expenses before starting the treatment processes (5.B).

Our findings declared that 73% of the students would rather refer the patient to the related specialist when they did not have the qualification or specialty for the treatment (2.B). Furthermore, 54.1% of the participants mentioned that they would cautiously treat the patients suffering from infectious diseases regarding the principles of infection control (4.A.1) and 53.2% of the students claimed that they would do all the necessary treatment procedures for the emergency patients (4.B).

According to the study findings, 51.4% of the students stated that they did not testify against their colleagues’ illegal or unethical performances to uphold their professional personality and did not even notify their colleagues of their wrong deeds. In this study, 71.2% of the participants stated that they would consult their colleagues if they faced an unfamiliar condition (2.B).

Results depicted that 73.9% of the study participants claimed that they would inform the patients if an infectious disease was transmitted to the patients during the treatment processes, and they would take the responsibility of subsequent follow up to provide necessary provisions (2.E). 

Furthermore, 75.7% of the students believed that they would treat the patients in a way that they could have their trust (2.G) and 60.4% of the students mentioned that before starting the treatment, they shared and explained all the possible treatment modalities to the patients and let them choose their own treatment plan (1.A).

Finally, in case of referring the patients to another dentist, 45.9% of the students stated that they would only pass the information which was necessary for the patients’ treatment to their colleagues (1.B).

## Discussion

Patients’ right is defined as what a treatment center is responsible to do for the patients. In other words, patients’ right is scrutinizing their legal, physical, psychological, spiritual, and social needs which are presented in treatment standards and regulations and the treatment team is responsible to monitor them [9].

The World Medical Association has recorded some issues as the guideline of care and treatment. In fact, without having knowledge about ethical concepts and their related issues, the health and treatment staff cannot challenge and concur with the demographic and technological changes of the 21^st^ century. Therefore, pa-ying attention to these concepts is of utmost importance to present health services with a desirable quality [10].

Akhondi Meybodi studied the knowledge of general practitioners as well as medical interns of Yazd province regarding the patients’ rights and observing them in the hospitals in 2007 and concluded that both the physicians and the interns were well informed about the patients’ rights but these rights were not strongly monitored in the hospitals. The results of the first part of the study were in line with those of the present research.

Nematollahi and Tofighi also compared the regulations of patients’ rights in Iran with the patients’ rights charter by surveying the physicians of Fars Province universities of medical sciences and health and treatment services. They revealed that no integrated set of patients’ rights existed in Iran [6]. However, in some parts of the laws of the ministry of health, treatment, and medical education, some regulations can be found regarding patient’s confidentiality, refusal of treatment, consultation on medical emergencies, and treatment expenditures invoice. Concerning medical emergencies, there are many similar laws which emphasize the patients’ right in receiving the health care services promptly. The laws also show the patients’ rights for confidentiality, but do not include the necessary details. Besides, the laws on consent do not account for informed consent. There are also some laws regarding refusal of treatment, respectful consultation, and treatment expenditures invoice; however, they do not involve the essential details. The lack of a compiled set of laws regarding the patients’ rights was confirmed by the findings of the current study, as well.

Furthermore, Salimi and Yarmohammadian concluded that the knowledge and observation of the patients’ rights charter regarding maintaining the patients’ dignity and observing the supportive performances were higher than the average level [11], which is consistent with the findings of the present research.

Finally, most of the students believed that they had knowledge about 60-70% of the medial ethics, which is in agreement with the students’ total score; i.e., 71%.

The results of the present study revealed no significant relationship between gender and the rate of monitoring the laws (*p*> 0.05). In addition, no significant relationship was found between gender and the importance of treatment expenditures (*p*> 0.05). Moreover, the total scores of the students entering the college in 2002, 2003, 2004, and 2005 were 0.228, 0.6226, 26.27, and 28.27 out of 38, respectively.

After all, since there are a great number of weak points in the patients’ rights charter which was developed and published by the ministry of health, treatment, and medical education, it is necessary to review the patients’ rights charters in other countries and establish a more reliable charter in Iran.

## Conclusion

Although the students’ awareness of the patients’ rights was at a desirable level (71%) in this study, awareness about some special rights, such as the principle of community service and the principles related to the patients’ certificates were not less than desirable which may represent the lack of a comprehensive rights charter in Iran.

Moreover, it is necessary to conduct more studies to change education of ethics in dentistry schools to reach the ultimate goals of the education. Also, by using other countries’ experiences and adjusting them with our religious and cultural beliefs to meet patients’ needs and rights. Integrated laws and regulations similar to commercial, international, administrative, and civil rights must be approved by the parliament and put into action by all public and private institutions. With rapid development of medical sciences, new issues arises which must take into account the mutual rights of the physicians, people, and patients. Development of such rights, in our country, requires performing scientific research based on the principles of Islam and concerning the academics of medical sciences. 
